# Reference-Assisted FBG Sensing for Simultaneous Carotid–Radial Pulse-Wave Measurement and PWV Estimation

**DOI:** 10.3390/s26216879

**Published:** 2026-10-30

**Authors:** Chang Hyun Park, Minju Jeong, Seongmun Jeong, Min Hur, Yoonji Lee, Hyung-Hoi Kim, Jeesu Kim, Chang-Seok Kim, Hwidon Lee

**Affiliations:** 1 Department of Cogno-Mechatronics Engineering, Pusan National University, Busan 46241, Republic of Korea; ckdgus9880@pusan.ac.kr (C.H.P.); minju010803@pusan.ac.kr (M.J.); smjeong99@pusan.ac.kr (S.J.); jeesukim@pusan.ac.kr (J.K.); ckim@pusan.ac.kr (C.-S.K.); 2 School of Transdisciplinary Engineering, Pusan National University, Busan 46241, Republic of Korea; hurmin04@pusan.ac.kr (M.H.); yunji24@pusan.ac.kr (Y.L.); 3 ARK Inc., Busan 46241, Republic of Korea; hhkim@pusan.ac.kr; 4 Engineering Research Center for Color-Modulated Extra-Sensory Perception Technology, Pusan National University, Busan 46241, Republic of Korea; 5 The Crystal Bank Research Institute, Pusan National University, Busan 46241, Republic of Korea

**Keywords:** fiber Bragg grating, pulse-wave sensing, pulse transit time, pulse wave velocity, reference-channel regression, carotid artery, radial artery

## Abstract

Simultaneous pulse-wave sensing at the carotid and radial arteries is challenging because the two sites require different mechanical coupling conditions and manually held carotid probes are susceptible to contact pressure and motion disturbances. This study presents a reference-assisted fiber Bragg grating (FBG) system comprising a wristband-type radial unit and a stamp-type carotid unit containing mechanically decoupled measurement and reference FBGs. Reference FBG regression (RFR) was used to suppress components shared by the carotid channels. In a 41.8 s recording from one male participant in his twenties, the reference and carotid measurement channels were essentially uncorrelated within the pulse-wave band (r = −0.05). After signal mean removal, RFR reduced power below 1 Hz by 74.3% without attenuating the pulse-band power (+11.4%), increasing the relative-power signal quality index from 0.174 to 0.458. Pulse transit time (PTT) was estimated by foot-centered windowed correlation. Analysis of 54 cardiac cycles yielded a representative PTT of 0.055 s. With an effective path length of 0.48 m, the corresponding pulse-wave velocity (PWV) was 8.7 m/s, with a within-record bootstrap 95% interval of 7.7–9.9 m/s. Estimates from eight non-overlapping 5 s segments were 9.1 ± 1.8 m/s. RFR produced lower segment-to-segment PTT variability than the baseline processing methods (coefficient of variation, 20.3% versus 34.6–46.7%). These results demonstrate the technical feasibility of reference-assisted FBG sensing for simultaneous carotid–radial pulse-wave acquisition and preliminary PWV estimation. Further validation requires higher-rate acquisition, repeated measurements, a larger cohort, and comparison with a reference PWV device.

## 1. Introduction

Pulse-wave velocity (PWV) is the velocity at which a pulse-wave propagates through the vascular system and has been investigated as an indicator associated with arterial stiffness and vascular function [1,2,3]. PWV is fundamentally calculated from the distance between two measurement sites and the difference in pulse arrival time [2,3]. However, in practice, the resulting value is affected by the selected measurement segment, the synchronization of the two signals, and the definition of the pulse-wave fiducial point [3,4,5]. Therefore, the measurement segment must be clearly defined when designing a PWV measurement system, and reproducible pulse-wave signals and timing information must be acquired reliably over that segment [6].

Conventional PWV and pulse-wave measurements use applanation tonometry, pressure or piezoelectric sensors, Doppler ultrasound, oscillometric cuffs, and photoplethysmography (PPG) [6,7,8,9,10]. For measurements performed at the body surface, changes in sensor position and coupling can alter pulse-wave morphology and amplitude. Simultaneous measurements at two anatomically distinct sites additionally require stable contact, synchronized acquisition, and scalable multichannel sensing [3,6,11]. These requirements motivated the use of fiber Bragg grating (FBG) sensors in this study.

An FBG sensor encodes external strain as a shift in the Bragg reflection wavelength of a grating formed within an optical fiber. FBGs offer compact size, wavelength-encoded output, immunity to electromagnetic interference, and multiplexing capability [12,13,14,15,16,17]. Accordingly, FBG and fiber-optic pressure sensors have been applied to biosignal monitoring, medical devices, biomechanical measurements, and wearable healthcare systems [11,18,19,20,21,22]. These properties are advantageous for synchronized optical measurements at spatially separated sites such as the carotid and radial arteries [14,18,19]. Previous studies have reported FBG-based pulse transit time measurement and local radial PWV measurement using flexible FBG sensors [23,24]. However, disturbance rejection in a manually held carotid FBG probe has received less attention.

The carotid–radial segment was selected because its two measurement sites impose different mechanical coupling conditions. A wristband can maintain stable fixation over the radial artery, whereas the carotid site is sensitive to neck curvature, respiration, posture, contact pressure fluctuations, and operator motion. A manually held probe can therefore capture both carotid pulse-induced deformation and probe-related disturbance, degrading waveform stability and timing analysis. To address this problem, the carotid unit incorporated a measurement FBG and a mechanically decoupled reference FBG. The reference channel was designed to capture variations transmitted through the probe handle with reduced sensitivity to local carotid deformation, allowing shared components to be suppressed by reference FBG regression (RFR) [25,26,27]. Previous FBG approaches differ from this configuration in both architecture and disturbance handling. Reference [23] acquires carotid and radial pulse waveforms with two separate FBG pulse recorders read on a single time scale, which removes the synchronization problem between two instruments; disturbance at the carotid site, however, is handled only by mechanical fixation of the probe. Reference [24] measures local PWV within the radial artery using two FBG units inscribed along each sensing fiber, one at the wrist and one at the fossa cubitalis, and no separate disturbance-reference channel was incorporated. The proposed system instead combines site-specific carotid and radial sensing units with a dedicated reference channel at the site where contact cannot be stabilized, and reads all three gratings from a single fiber and a single photodetector trace so that the channels share one time base.

## 2. Methods

### 2.1. FBG Sensor System

**Figure 1:**
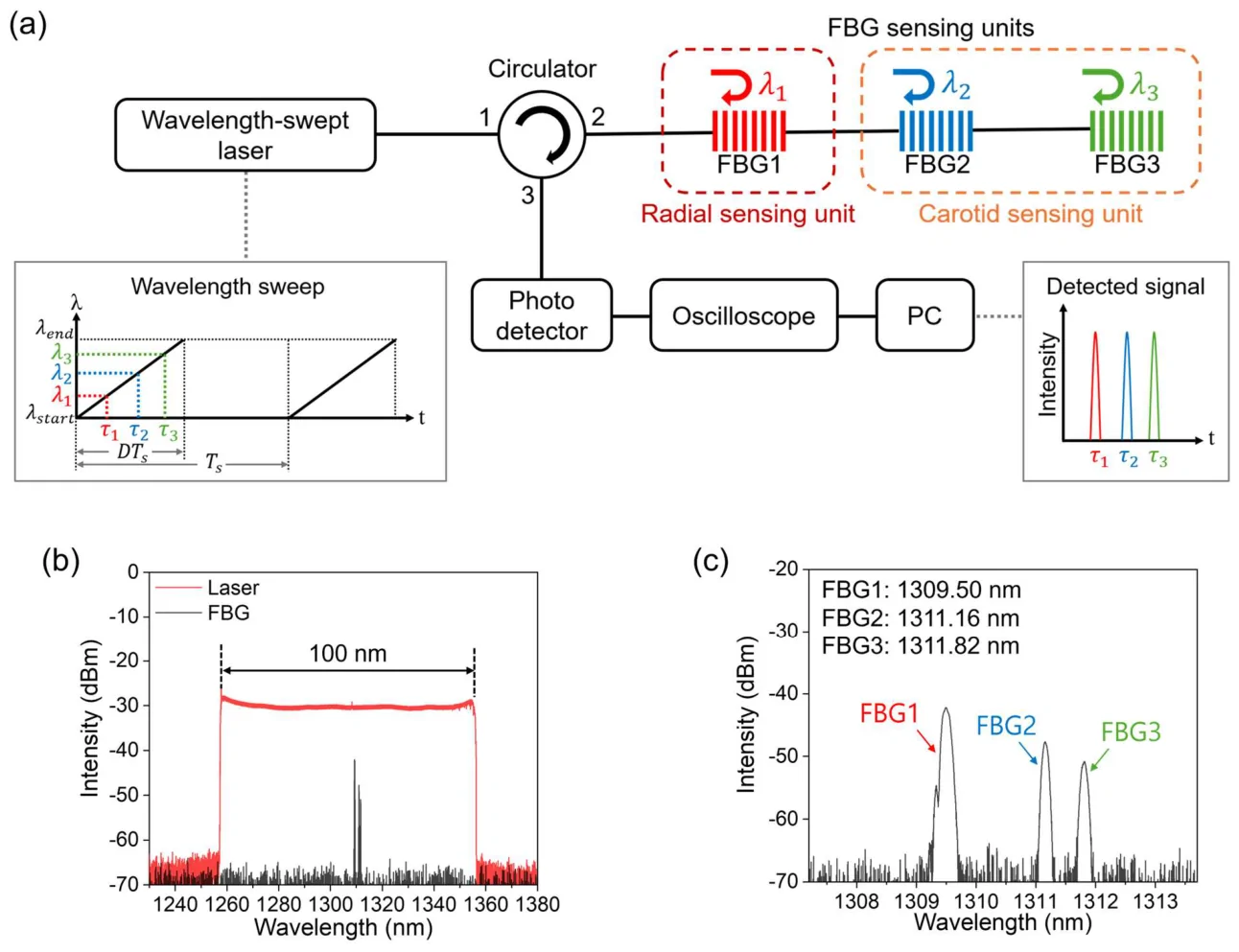
Configuration and spectral characteristics of the FBG sensor system: (**a**) system configuration; (**b**) laser output spectrum and reflection spectra of the three FBGs; (**c**) enlarged view of the FBG reflection spectra.

Figure 1 shows the configuration and spectral characteristics of the FBG sensor system developed in this study. As shown in Figure 1a, light emitted from the wavelength-swept laser was directed through an optical circulator to the FBG sensing units in which the three FBGs were connected in series along a single optical fiber. The radial sensing unit contained FBG1, and the carotid sensing unit contained FBG2 and FBG3. The light reflected from the FBGs was then routed through the circulator to a photodetector, and the resulting signal was recorded with an oscilloscope (RTO64, Rohde & Schwarz, Munich, Germany).

The recorded waveforms were transferred to a computer for subsequent processing, and the transfer rate limited the pulse-wave acquisition to a mean rate of 13.2 Hz. Because the transfer was not hardware-triggered, the acquisition instants were not strictly uniform (mean interval 0.076 s, SD 0.006 s, range 0.067–0.127 s); the time stamp of every frame was recorded, so the irregular sampling instants were retained and taken into account in the subsequent resampling. Figure 1b shows the laser output spectrum and the reflection spectra of the three FBGs, and Figure 1c shows an enlarged view of the reflection spectra. The laser had a sweep repetition rate of 50 kHz, a duty cycle of 50%, and a tuning range of 100 nm. Its instantaneous linewidth was not measured independently in this study. The manufacturer specifies a coherence length of 28 mm; because the conversion from coherence length to linewidth depends on the fall-off definition and on the assumed spectral line shape, the corresponding linewidth is treated here as an estimate, and is approximately 0.03 nm for a Gaussian line shape with the 50% coherence length convention and approximately 0.06 nm for the simpler λB^2^/Lc relation. Either value is well below the measured 3 dB reflection bandwidths of the gratings reported in Section 2.3. The center wavelengths of FBG1, FBG2, and FBG3 were 1309.50, 1311.16, and 1311.82 nm, respectively, and all three lay within the tuning range of the laser. Because the reflection peaks of the FBGs were located at different wavelengths, they were detected at different times within each wavelength sweep, which allowed the three FBGs to be read independently from a single photodetector output.

An FBG selectively reflects a specific wavelength component, and its center reflection wavelength is defined as the Bragg wavelength, $\lambda_{B}$. The Bragg wavelength is expressed in terms of the effective refractive index of the fiber core, $n_{eff}$, and the grating period, Λ, as given in Equation (1) [12,13].

(1) $$\lambda_{B}=2n_{eff}\Lambda .$$

When the sensing unit is subjected to external strain or a temperature change, the effective refractive index $n_{eff}$, the grating period Λ, or both change, resulting in a shift in the Bragg wavelength of the FBG [14,15]. Thus, the Bragg wavelength shift represents the FBG response to strain or temperature variations experienced by the sensing unit. Instead of directly converting the shift into wavelength units, this study quantified it based on changes in the within-sweep timing of the reflection peak detected in the photodetector output during repeated wavelength sweeps of the laser.

The FBG sensor system with a wavelength-swept laser utilizes the time-dependent variation in the instantaneous laser output wavelength during each wavelength sweep. As the laser repeatedly sweeps over a predefined wavelength range, each time point within a sweep corresponds to a specific output wavelength. A reflected-light peak occurs when the laser output wavelength coincides with the Bragg wavelength of an FBG. Therefore, a shift in the Bragg wavelength causes a corresponding shift in the time at which the reflection peak is detected within the sweep. Assuming a linear wavelength sweep, the laser output wavelength at an elapsed time τ from the start of a sweep can be expressed as Equation (2),

(2) $$\lambda_{L}\left(\tau \right)=\lambda_{start}+k_{\lambda }\tau ,\;\;\;\;\;\;0\leq \tau \leq {DT}_{s},$$

where $\lambda_{L}$ is the instantaneous laser output wavelength, $\lambda_{start}$ is the wavelength at the beginning of a sweep, $T_{s}$ is the sweep repetition period, D is the duty cycle, and $k_{\lambda }$ is the wavelength sweep rate. The wavelength sweep rate is defined by Equation (3)

(3) $$k_{\lambda }=\frac{\lambda_{end}-\lambda_{start}}{{DT}_{s}},$$

where $\lambda_{end}$ is the wavelength at the end of a sweep. With $T_{s}$ = 20 μs and D = 0.5 for the laser used in this study, $k_{\lambda }$ was approximately 10 nm/μs. This value is the average sweep slope over the full tuning range and is the quantity used in Equation (5).

The within-sweep detection times of the FBG reflection peaks were tracked from the photodetector output over repeated wavelength sweeps. Because each peak time corresponds to the Bragg wavelength of an FBG, a shift in the Bragg wavelength produces a corresponding shift in the peak time. Let $\tau_{p}\left(t\right)$ denote the within-sweep peak time at measurement time t and let $\tau_{p,0}$ denote the corresponding peak time in the reference state. The peak time shift $\Delta \tau_{p}\left(t\right)$ relative to the reference state is defined by Equation (4).

(4) $$\Delta \tau_{p}\left(t\right)=\tau_{p}\left(t\right)-\tau_{p,0}.$$

Under the assumption of a linear wavelength sweep, the Bragg wavelength shift and the peak time shift are related by Equation (5).

(5) $$\Delta \tau_{p}\left(t\right)=\frac{\lambda_{B}\left(t\right)-\lambda_{B,0}}{k_{\lambda }}.$$

Here, $\lambda_{B}\left(t\right)$ and $\lambda_{B,0}$ denote the Bragg wavelengths at measurement time t and in the reference state, respectively. In this study, the Bragg wavelength shift was not explicitly calculated; instead, the corresponding within-sweep peak time shift, $\Delta \tau_{p}\left(t\right)$, was used as the measured quantity.

### 2.2. Characterization of the FBG Sensor

A static strain test was conducted to characterize the strain response of the FBG sensor system. A 60 cm optical fiber test section containing the FBG was mounted on a strain-application stage, with a free span of 7.5 cm between the two fixing points, and tensile strain was applied incrementally by increasing the stage displacement in 5 μm steps. Assuming uniform deformation over the 7.5 cm span under tension, each displacement step corresponded to a strain increment of approximately 66.7 με. Sixteen strain levels were measured, comprising an unstrained reference state and 15 successive increments, giving a maximum applied strain of 1000 με. At each strain level, the within-sweep peak time was tracked continuously for 5 s, and the peak time shift $\Delta \tau_{p}$ relative to the reference state was measured.

**Figure 2:**
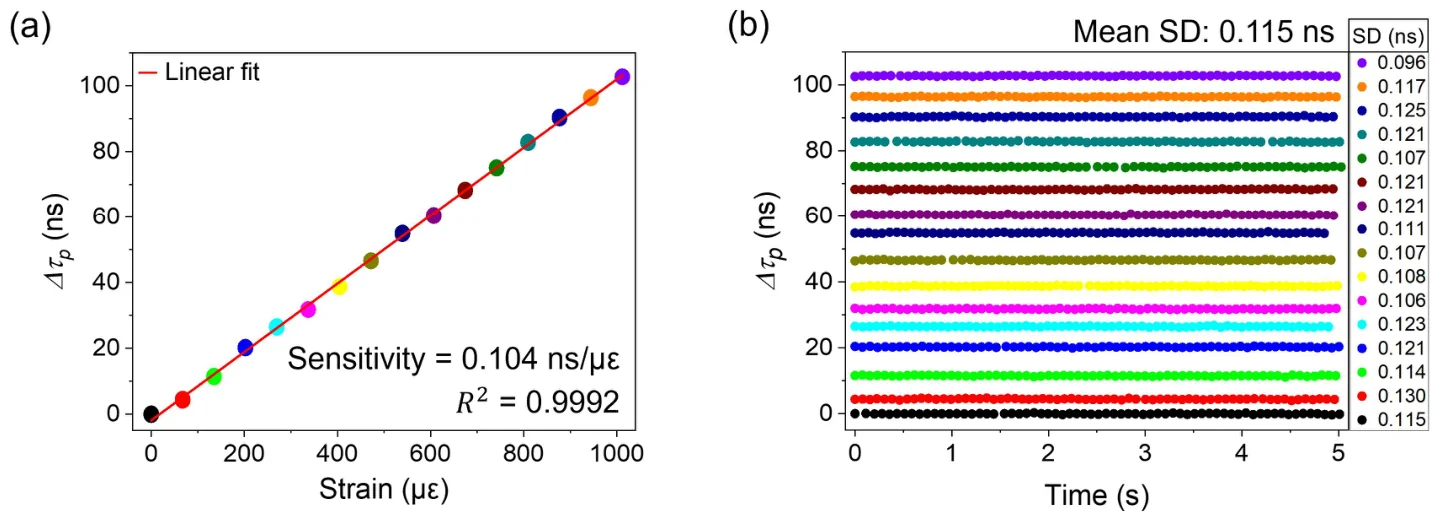
Static strain test results, color-coded by strain level in both panels: (**a**) peak time shift $\Delta \tau_{p}$ as a function of the applied strain, with the linear fit; (**b**) $\Delta \tau_{p}$ recorded continuously over 5 s at each strain level, with the standard deviation (SD) of each level listed in the legend.

The results of the static strain test are presented in Figure 2. Figure 2a shows the relationship between the peak time shift and applied strain over approximately 0–1000 με, together with the linear regression fit. The coefficient of determination, R^2^, was 0.9992, and the sensitivity determined from the regression slope was 0.104 ns/με. Figure 2b shows the repeated peak time shift measurements acquired for 5 s at each strain level. The mean of the standard deviations across the strain levels was 0.115 ns, corresponding to an equivalent strain standard deviation of 1.11 με. The corresponding two-standard-deviation value was 2.22 με. Through the sweep rate given by Equation (3), this sensitivity corresponds to a wavelength sensitivity of 1.04 pm/με, consistent with the value of approximately 1.02 pm/με expected from (1 − *p*_e_)*λ*_B_ at 1310 nm, and the mean standard deviation of 0.115 ns corresponds to a short-term wavelength-equivalent standard deviation of approximately 1.15 pm. This value characterizes the short-term repeatability of the peak time estimate under the present experimental conditions, including sweep repeatability, peak-detection noise, and electronic noise, rather than the absolute wavelength accuracy of the interrogation. For reference, the reflection peak in the photodetector trace is estimated to be approximately 12–17 ns wide, obtained by combining the measured 3 dB reflection bandwidth and the estimated laser linewidth in quadrature and dividing by the sweep rate of Equation (3), so the peak time standard deviation is about two orders of magnitude smaller than the width of the peak whose center is being located.

### 2.3. Configuration of the FBG Sensing Units

**Figure 3:**
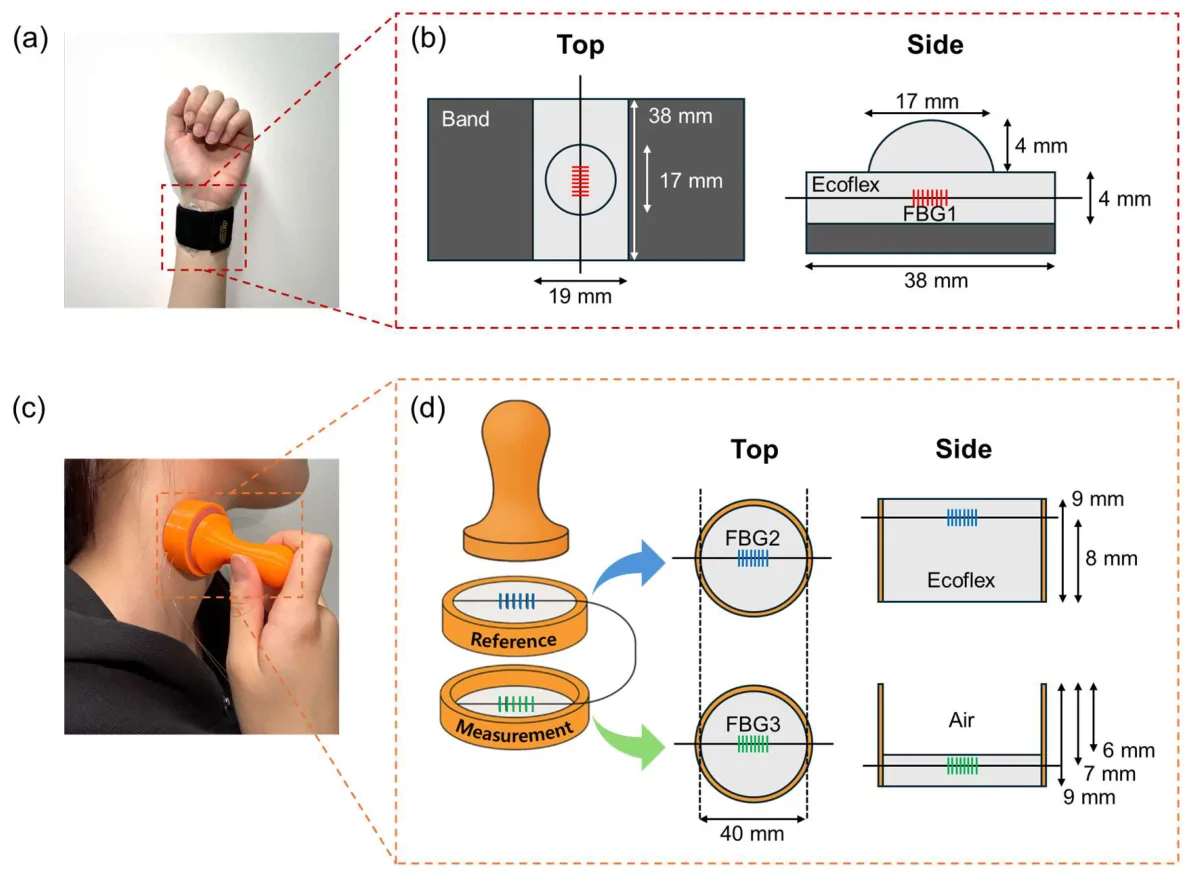
FBG sensing units: (**a**) the wristband-type radial sensing unit in place on the wrist; (**b**) top and side views of its contact element; (**c**) the stamp-type carotid sensing unit in place on the neck; (**d**) top and side views of its reference and measurement parts.

Figure 3 shows the FBG sensing units used in this study. All three FBGs were embedded in a soft silicone elastomer (Ecoflex 00-50, Smooth-On, Inc., Macungie, PA, USA). Each FBG had a grating length of 10 mm. The 3 dB reflection bandwidths measured from the spectra in Figure 1c were 0.16, 0.12 and 0.13 nm for FBG1, FBG2 and FBG3, respectively, and were well below the minimum separation of 0.66 nm between adjacent FBG peaks. The fiber was fully embedded during casting of the elastomer, so that it was mechanically coupled to the elastomer along its embedded length without additional adhesive. A small pre-strain was applied to keep the fiber taut during encapsulation; its magnitude was not quantified, and the linearity of the strain response over the range characterized in Section 2.2 was confirmed after fabrication. Figure 3a shows the wristband-type sensing unit for radial artery measurement worn on the wrist, and Figure 3b shows its contact element. The sensing unit included a contact element consisting of a rectangular base (38 mm × 19 mm × 4 mm) with a dome-shaped protrusion (17 mm in diameter and 4 mm in height), which transmitted local deformation over the radial artery to FBG1. Figure 3c shows the stamp-type sensing unit for carotid artery measurement positioned with its measurement FBG in contact with the carotid pulsation, and Figure 3d shows its configuration of reference and measurement parts, which comprises a measurement FBG in contact with the skin, a reference FBG in direct contact with the handle, and an air gap that mechanically isolates the two sensing regions. Each part was 40 mm in diameter and 9 mm thick and contained one FBG: FBG2 as the reference FBG and FBG3 as the measurement FBG.

In the wristband-type sensing unit, band tension maintained contact between the dome-shaped protrusion and the wrist surface over the radial artery, allowing surface deformation induced by radial artery pulsation to be transmitted through the contact element to the FBG sensing region. Compared with a planar contact surface, the dome-shaped protrusion provided a smaller and more localized contact area through which the surface deformation was transmitted.

In the stamp-type sensing unit, the measurement FBG was positioned against the skin over the carotid artery, whereas the reference FBG was coupled to the handle rather than the skin. An air gap between the two FBG regions mechanically decoupled them, allowing the measurement FBG region to deform more readily while suppressing direct transmission of carotid pulse-induced deformation to the reference FBG. This configuration allowed the measurement FBG to detect local surface deformation induced by carotid artery pulsation, whereas the reference FBG primarily detected common-mode disturbances transmitted through the handle, including changes in contact pressure, small hand tremors, respiration-related motion, and ambient vibration, with reduced sensitivity to local carotid pulsation. The degree of this asymmetry is quantified in Section 2.4.2: within the pulse-wave band, the reference and measurement channels are essentially uncorrelated, whereas across the full band, a substantial part of their variance is shared, which is the condition that RFR requires.

### 2.4. Pulse-Wave Measurement and Analysis

#### 2.4.1. Measurement Protocol and Signal Acquisition

A single male participant in his twenties, who is one of the authors, remained seated throughout the measurement with the head in a neutral position. The wristband-type sensing unit was secured over the radial artery, whereas an operator held the stamp-type sensing unit against the skin over the common carotid artery. Both sites were measured on the same side of the body. Contact pressure was not actively controlled; its variation was treated as a common-mode disturbance targeted by RFR rather than as a calibrated input. The two pulse-wave signals were acquired simultaneously for 41.8 s at a mean rate of 13.2 Hz, yielding 553 samples.

#### 2.4.2. Signal Processing and Reference FBG Regression

The carotid and radial pulse-wave signals were acquired at irregular instants at a mean rate of 13.2 Hz and were resampled onto a common uniform time axis at 0.005 s intervals using cubic spline interpolation. Because all three FBGs were read from the same photodetector trace, they shared a common acquisition time base and resampling introduced no relative timing offset among the channels. The uniform grid allowed correlation coefficients to be evaluated at common time points and candidate fractional shifts to be tested in 0.005 s increments. This numerical interpolation did not increase the intrinsic temporal resolution of the 13.2 Hz acquisition or constitute acquisition at 200 Hz.

For noise reduction, the carotid measurement FBG signal (y(t)) was expressed as the sum of a pulse-wave component and a disturbance component, as given in Equation (6) [25]:

(6) y(t)=s(t)+n(t).

Here, s(t) is the pulse-wave component induced by carotid pulsation, and n(t) is the disturbance component arising from contact pressure variations, sensor motion, respiration-related motion, ambient vibration, and other external factors.

As shown in Figure 3d, the reference FBG was configured to reduce its direct response to carotid pulsation and primarily capture disturbances common to the two FBG channels. The signal acquired from the reference FBG was denoted by r(t).

In this study, a linear regression analysis was performed using IBM SPSS Statistics 26, with the reference FBG signal r(t) as the regressor and the measurement FBG signal y(t) as the dependent variable. The disturbance $\hat{n}\left(t\right)$ estimated to be common to the two channels was obtained using RFR, an implementation of the reference channel regression method [25,26,27], as given in Equation (7):

(7) $$\hat{n}\left(t\right)={\hat{\beta }}_{0}+{\hat{\beta }}_{1}r\left(t\right).$$

Here, ${\hat{\beta }}_{0}$ and ${\hat{\beta }}_{1}$ denote the estimated intercept and slope coefficient relating r(t) to y(t). The noise-reduced carotid signal $y_{NR}\left(t\right)$ was obtained by subtracting the estimated common disturbance $\hat{n}\left(t\right)$ from the measurement FBG signal y(t), as given in Equation (8):

(8) $$y_{NR}\left(t\right)=y\left(t\right)-\hat{n}\left(t\right).$$

For the 41.8 s recording analyzed in this study, the regression of the measurement FBG signal on the reference FBG signal gave an intercept of 1.91 ns and a slope of 0.856, with a coefficient of determination of 0.583 (Pearson r = 0.764), indicating that a substantial but not dominant fraction of the measurement FBG variance was shared with the reference channel. Within the 1–2.25 Hz pulse-wave band, by contrast, the band-pass-filtered reference and measurement signals were essentially uncorrelated (r = −0.05), whereas the band-pass-filtered carotid and radial signals were strongly correlated (r = 0.937). The magnitude-squared coherence between the carotid and reference channels at the heart rate fundamental (1.32 Hz) was 0.24, compared with 0.97 between the carotid and radial channels. These observations are consistent with reduced direct transmission of carotid pulsation to the reference FBG and support the use of RFR to suppress shared disturbance while preserving the pulse-wave component.

To quantitatively evaluate the noise-reduction effect of RFR, FFT-based power spectra of the carotid signals before and after RFR were compared. The relative-power-based signal quality index proposed for PPG signal assessment was applied exploratorily to the FBG pulse-wave signal [28]. Before spectral calculation, the arithmetic mean of each 41.8 s signal was subtracted to remove the arbitrary constant offset associated with the initial Bragg wavelength and static strain. Mean subtraction was performed before windowing because a nonzero DC level can be redistributed into adjacent low-frequency bins when a nonrectangular window is applied [29]. A Welch window was then applied to each demeaned signal, and the one-sided FFT-based power spectrum was calculated in OriginPro and expressed in terms of mean squared amplitude (MSA). Note that [28] estimates spectra using Welch’s averaged-periodogram method, whereas a single-segment estimate was used here to retain the 0.024 Hz frequency resolution required to separate the slow-variation and pulse-wave bands. No linear detrending or high-pass filtering was applied in this spectral comparison; thus, slow variations targeted by RFR remained within the denominator. The pulse-wave band was set to 1–2.25 Hz following the previous study, whereas the overall analysis band was set to 0–6 Hz considering the Nyquist frequency of 6.6 Hz associated with the mean 13.2 Hz acquisition rate. Based on these bands, the index *R*_SQI_ was defined as Equation (9):

(9) $$R_{SQI}=\frac{\int_{1}^{2.25}P\left(f\right)\;df}{\int_{0}^{6}P\left(f\right)\;df}.$$

Here, P(f) denotes the FFT-based power spectrum at frequency f, expressed in terms of MSA.

To determine whether the reference channel provided information unavailable from the measurement channel alone, RFR was compared with three baseline methods applied to the same carotid signal: subtraction of a ninth-order polynomial trend and second-order Butterworth high-pass filtering with cut-off frequencies of 0.5 and 0.8 Hz. The high-pass filters were applied in a forward–backward, zero-phase configuration, so that no filter-induced phase delay could enter the timing estimates; pulse-wave feet were detected once, on the RFR-processed carotid signal, and the resulting 54 foot positions and analysis windows were applied unchanged to every processing method, so that the comparison differs only in the preprocessing of the carotid signal. For each method, the signal quality index was recalculated after the same demeaning and windowing, and the peak correlation coefficient, representative PTT and PWV, and coefficient of variation of the PTT estimates from eight non-overlapping 5 s segments were calculated using the same time shift analysis described in Section 2.4.3. These comparisons were descriptive and were not treated as independent measurements or inferential tests.

#### 2.4.3. PTT Estimation and PWV Calculation

The PTT between the carotid and radial arteries was estimated around the foot, defined as the onset of the pulse-wave upstroke and selected because it is generally less affected by wave reflection and waveform distortion than the peak [3,4,5]. Every analyzable cardiac cycle in the 41.8 s recording was included. Local minima of the RFR-processed carotid signal identified 54 usable cycles with a mean interbeat interval of 0.763 s (SD, 0.044 s), corresponding to a mean heart rate of 78.7 beats/min. A 0.3 s analysis window was defined for each cycle, beginning 0.12 s before the carotid foot and ending 0.18 s after it. This window ended before the carotid systolic peak, which occurred 0.26 s (SD, 0.06 s) after the foot; longer windows include portions whose morphology differs between the carotid and radial sites and can bias the estimated delay. Sensitivity to window offsets from −0.20 to 0 s and lengths from 0.20 to 0.40 s is reported in Figure S1 of the Supplementary Materials. The RFR-processed carotid and radial signals were independently min–max normalized over the full recording. Candidate shifts from −0.10 to 0 s were applied to the radial signal in 0.005 s increments, where a negative shift advanced the radial waveform. Pearson correlation coefficients were calculated within each window and then averaged across all 54 windows. A Gaussian function was fitted to the mean correlation curve, and the absolute shift at its fitted peak was taken as the representative PTT. A within-record 95% bootstrap interval was obtained by resampling the cycles with replacement for 3000 replicates and repeating the averaging and Gaussian fitting. Within-record stability was assessed by applying the same procedure to eight non-overlapping 5 s segments. As a correlation-independent check, the maximum first derivative of each carotid and radial pulse was identified using a Savitzky–Golay differentiator, and the median cycle-wise delay was compared with the correlation-based estimate. PWV was calculated from the representative PTT and effective path length L using Equation (10) [2,3]:

(10) $$PWV=\frac{L}{PTT}.$$

The effective path length L was determined using the surface-distance subtraction method [2,3,5]. The suprasternal-notch-to-carotid distance of 0.14 m was subtracted from the suprasternal-notch-to-radial distance of 0.62 m, both measured using a tape measure, resulting in L = 0.48 m.

## 3. Results

### 3.1. Simultaneous Carotid–Radial Pulse-Wave Measurement

**Figure 4:**
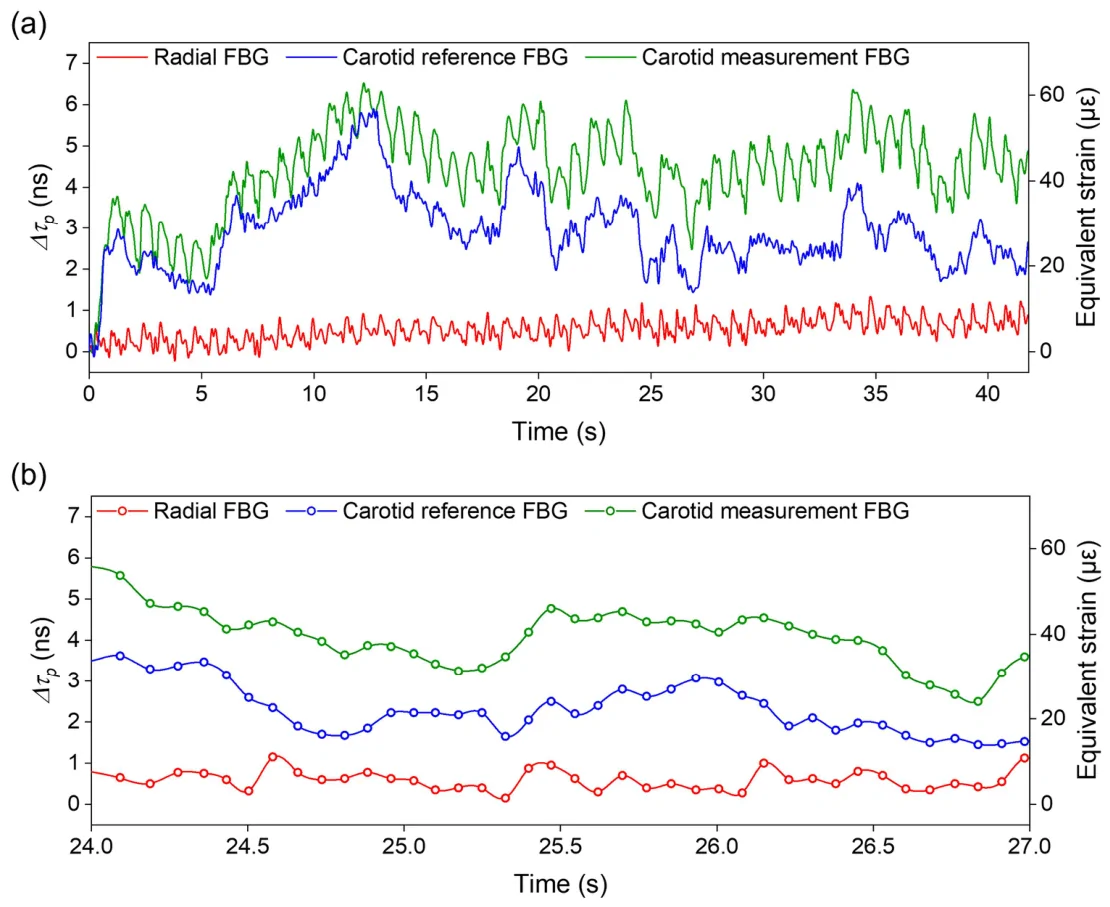
Simultaneous carotid and radial pulse-wave measurements: (**a**) measured $\Delta \tau_{p}$ signals from the radial FBG, the carotid reference FBG, and the carotid measurement FBG before reference FBG regression (RFR). The right-hand axis gives the equivalent strain obtained from the sensitivity of 0.104 ns/με; (**b**) enlarged view of the 24–27 s interval, in which open circles mark the native acquisition instants and the lines show the cubic spline used for resampling onto the uniform 0.005 s grid. The 0.005 s interpolation grid was used only for numerical fractional-shift evaluation and does not represent an increased acquisition rate.

Figure 4a shows the peak time shifts $\Delta \tau_{p}$ simultaneously acquired from the radial FBG, the carotid measurement FBG before RFR, and the carotid reference FBG. Periodic pulse-wave patterns corresponding to the cardiac cycle were observed in the radial and carotid measurement FBG signals. In contrast, the carotid reference FBG signal primarily exhibited variations attributed to changes in contact pressure and sensor motion. This signal was subsequently used as the reference input for estimating disturbance components common to the two carotid FBG channels. Figure 4b shows an enlarged 3 s interval in which the native acquisition instants are plotted together with the cubic spline used for resampling; the mean acquisition interval of 0.076 s is comparable to the width of the systolic upstroke, which is the reason why the transit time was estimated from an ensemble of cycles rather than from individual fiducial points.

Using the strain sensitivity of 0.104 ns/με obtained in Section 2.2, the peak-to-peak pulse-wave amplitudes correspond to equivalent strains of 10.6 με at the carotid site and 5.8 με at the wrist, against an equivalent strain noise of 1.11 με determined from the static test. Over the whole recording, the carotid reference FBG signal spanned 58.1 με, approximately five times larger than the pulse-wave amplitude itself, which illustrates the magnitude of the disturbance that the reference channel was intended to capture.

### 3.2. Carotid Signal Noise Reduction

**Figure 5:**
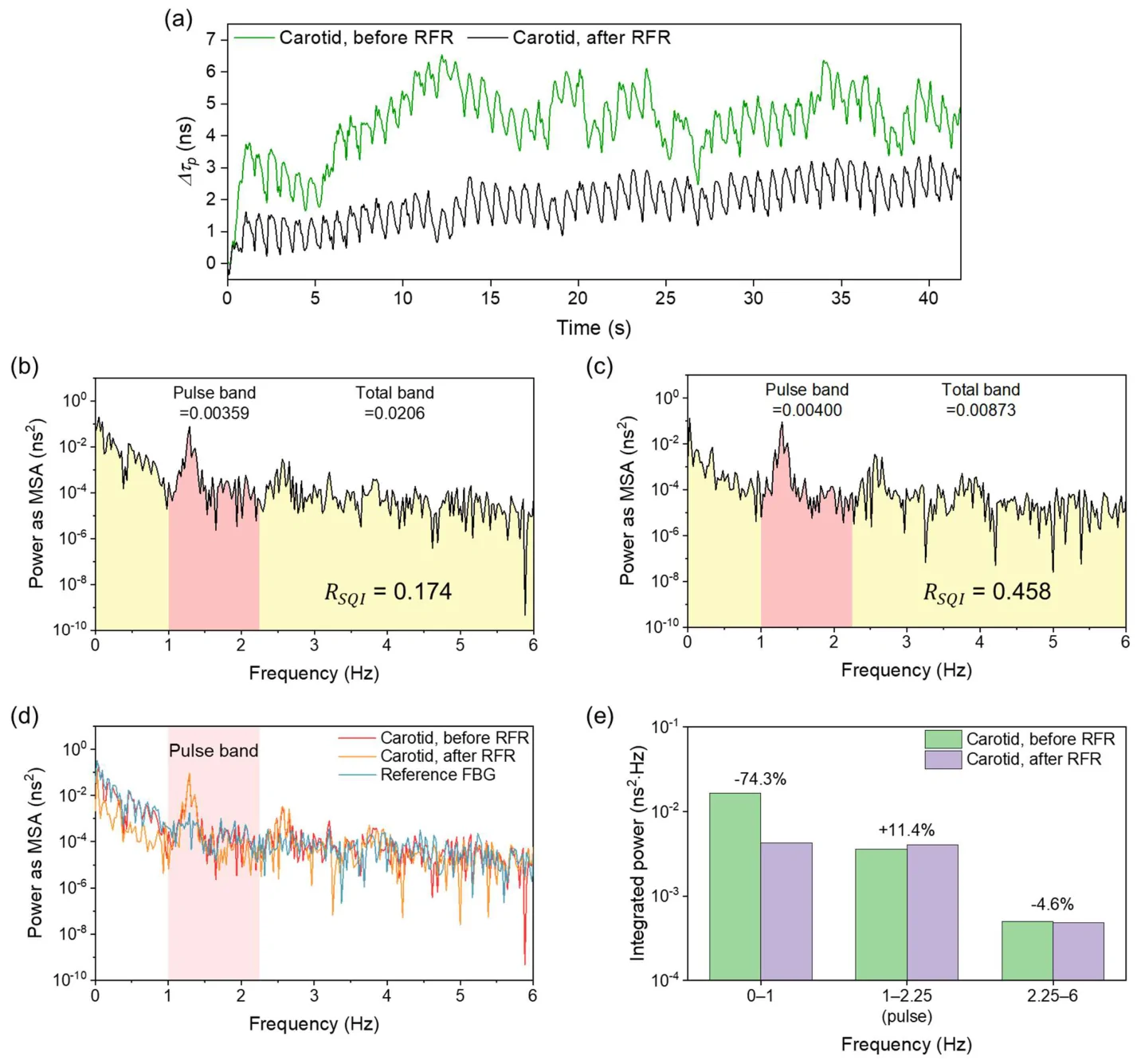
Time- and frequency-domain comparison of the carotid peak time shift signals before and after reference FBG regression (RFR): (**a**) time series before and after RFR; (**b**) FFT-based power spectrum before RFR; (**c**) FFT-based power spectrum after RFR; (**d**) power spectra of the carotid signal before and after RFR together with that of the reference FBG, with the 1–2.25 Hz pulse-wave band shaded; (**e**) integrated power in the 0–1, 1–2.25, and 2.25–6 Hz bands before and after RFR. Before calculating the spectra, the arithmetic mean of each 41.8 s signal was subtracted and a Welch window was applied.

RFR was applied to the carotid measurement FBG signal to reduce variations common to the two FBG channels. Figure 5a compares the carotid peak time shifts $\Delta \tau_{p}$ before and after RFR. Before RFR, large slow variations were superimposed on the periodic pulse-wave component. After RFR, these variations were reduced, making the periodic pulse-wave component more distinct.

Figure 5b,c show the FFT-based power spectra of the demeaned carotid signals before and after RFR, respectively. Before RFR, the integrated powers in the 1–2.25 Hz pulse-wave band and over the 0–6 Hz analysis band were 0.00359 and 0.0206 ns^2^·Hz, respectively, resulting in *R*_SQI_ = 0.174. After RFR, the corresponding values were 0.00400 and 0.00873 ns^2^·Hz, and *R*_SQI_ increased to 0.458. The power below 1 Hz decreased by 74.3%, whereas the pulse-band power increased by 11.4% and the power between 2.25 and 6 Hz decreased by 4.6%. The total 0–6 Hz power consequently decreased by 57.6%, and *R*_SQI_ increased by a factor of 2.63. These results indicate that the effect of RFR was concentrated primarily in the low-frequency variations rather than in broad-band attenuation. The increase in pulse-band power is consistent with the addition of variance that is uncorrelated with the carotid pulse, although band power alone does not establish that the pulse waveform is preserved: the product of the squared regression slope and the pulse-band power of the reference channel corresponds to +8.1% of the pulse-band power before RFR, close to the observed +11.4%. Figure 5d compares the three power spectra, and Figure 5e summarizes the band-wise power changes. The same spectral analysis applied to the reference FBG signal gave a signal quality index of 0.015, confirming that this channel carried little pulse-wave content, whereas the RFR-processed carotid signal reached 0.458, exceeding the 0.392 obtained for the mechanically fixed wristband radial channel. Waveform morphology was assessed separately by comparing the cycle-ensemble carotid waveform, obtained by averaging all 54 cycles about the detected foot, before and after RFR. The correlation between the two ensemble waveforms was 0.998, the root-mean-square difference after amplitude normalization was 0.023 of the unit pulse amplitude, and the foot-to-peak interval changed from 0.250 to 0.255 s. This supports, but does not by itself establish, preservation of the pulse waveform.

Table 1 compares RFR with the baseline methods described in Section 2.4.2. The five processing conditions gave whole-record PTT estimates of 0.047–0.055 s (PWV, 8.7–10.2 m/s), a spread comparable to the within-record bootstrap interval of 0.048–0.062 s obtained for RFR. The two high-pass baselines reached higher values of the signal quality index than RFR (0.74 and 0.82 versus 0.46), because the index rewards any operation that removes low-frequency power irrespective of whether the timing information is improved. The downstream comparison gave the opposite ordering: RFR yielded the highest peak correlation coefficient (0.896 versus 0.843–0.870) and the lowest segment-to-segment variability of the PTT estimate (coefficient of variation, 20.3% versus 34.6–46.7%). This comparison connects the reference-assisted reduction in slow disturbance to improved stability of the waveform alignment used for PTT estimation.

### 3.3. PTT Determination and PWV Estimation

**Figure 6:**
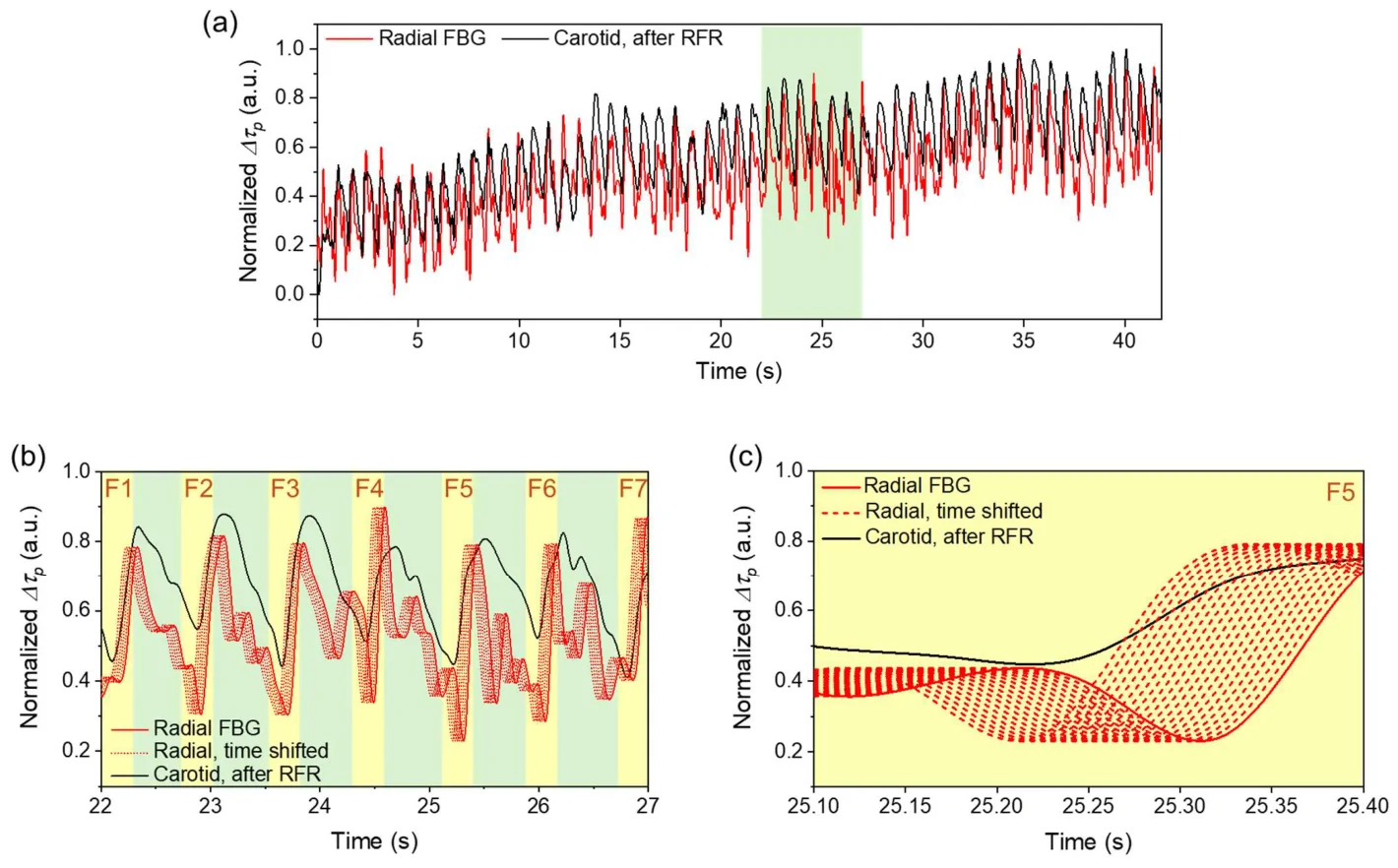
Time shift analysis for carotid–radial pulse transit time: (**a**) analysis interval indicated by green shading, (**b**) seven of the 0.3 s analysis windows (F1–F7) indicated by yellow shading, and (**c**) enlarged view of F5 showing waveform alignment at different time shifts.

Figure 6 shows the time shift analysis used to determine the carotid–radial PTT, with the 22–27 s interval of the recording used as a representative excerpt. The green-shaded region in Figure 6a indicates this interval. Figure 6b provides an enlarged view of it, where the yellow-shaded regions, denoted F1–F7, are seven consecutive analysis windows out of the 54 windows defined over the whole recording. Within each window, the candidate time shifts were applied to the radial signal, and the alignment of the shifted radial waveform with the RFR-processed carotid waveform was evaluated. Figure 6c provides an enlarged view of F5 and shows how this alignment varies with the applied time shift.

**Figure 7:**
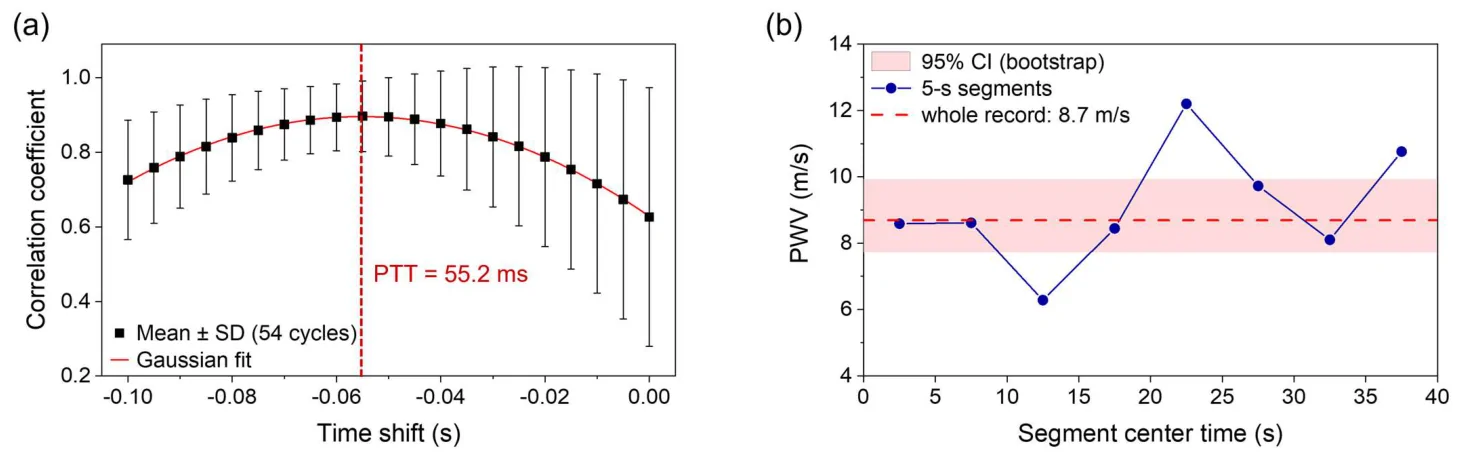
Carotid–radial transit time analysis: (**a**) mean of the carotid–radial correlation coefficients calculated across all 54 analysis windows as a function of the applied time shift, together with the fitted Gaussian function and the SD of the coefficients; (**b**) PWV estimated from eight non-overlapping 5 s segments of the recording, with the whole-record estimate and its within-record bootstrap 95% interval indicated.

Figure 7a shows the mean and standard deviation of the carotid–radial correlation coefficients calculated across all 54 analysis windows at each candidate time shift, together with the fitted Gaussian function. The peak of the fitted Gaussian was located at a time shift of −0.055 s, with a fitted peak correlation of 0.90. Because a negative time shift denotes advancement of the radial signal, its absolute value, 0.055 s, was taken as the representative PTT from the carotid to the radial artery. Using the measured distance L = 0.48 m, the resulting PWV was 8.7 m/s.

Resampling the 54 cycles with replacement produced a within-record 95% bootstrap interval of 0.048–0.062 s for the representative PTT, corresponding to 7.7–9.9 m/s for PWV. Applying the same procedure to eight non-overlapping 5 s segments yielded PWV estimates of 6.3–12.2 m/s, with a mean of 9.1 m/s and an SD of 1.8 m/s (PWV coefficient of variation, 19.8%). The corresponding coefficient of variation of the segment-wise PTT values was 20.3%, as reported in Table 1. The independent maximum-slope analysis detected 55 pulse pairs, one more than the 54 correlation windows because the first cycle of the recording did not allow a complete analysis window to be placed before its foot, and yielded a median delay of 0.058 s, corresponding to 8.3 m/s and supporting the correlation-based estimate. Direct cycle-wise foot-to-foot delays were −0.007 ± 0.088 s, centered near zero with large dispersion. Thus, single-cycle foot timing was not usable at the present acquisition rate, and ensemble averaging was required.

## 4. Discussion and Conclusions

This study demonstrated a reference-assisted FBG configuration for simultaneous pulse-wave sensing at mechanically dissimilar carotid and radial sites. The wristband-type radial unit maintained fixed contact at the wrist, whereas the handheld carotid unit incorporated a measurement FBG and a mechanically decoupled reference FBG. The reference channel was not assumed to contain only disturbance; instead, it was designed to be less sensitive to local carotid deformation and to capture variations transmitted through the probe handle. Accordingly, RFR should be interpreted as suppression of components shared by the two carotid channels rather than isolation of a physiologically pure carotid waveform. After removal of the constant signal mean, RFR reduced power below 1 Hz by 74.3%, while the pulse-band power was not attenuated (+11.4%), an increase consistent with the addition of variance uncorrelated with the carotid pulse. The reduction in slow disturbance is directly demonstrated by these band power changes, whereas the evidence for preservation of the pulse waveform is indirect: the cycle-ensemble carotid waveform was essentially unchanged by RFR (correlation 0.998; normalized root-mean-square difference 0.023; foot-to-peak interval 0.250 versus 0.255 s). A conclusive assessment of morphological fidelity would require simultaneous comparison with a reference pressure waveform, which was not available here. The resulting increase in the relative-power signal quality index from 0.174 to 0.458 therefore primarily reflects suppression of slow disturbance. RFR also produced the highest whole-record correlation and the lowest segment-wise PTT variability among the evaluated processing methods, directly connecting the reference-assisted disturbance reduction to the waveform alignment used for PTT estimation.

After reducing non-pulse-wave variations in the carotid signal through RFR, PTT analysis was performed using the RFR-processed carotid signal and the radial signal. In this analysis, directly determining the time difference between the carotid and radial feet within a single pulse-wave cycle can be sensitive to local noise and uncertainty in foot identification; in the present recording, the direct delays were centered near zero with large dispersion. Therefore, to incorporate the waveform information around the pulse-wave feet rather than relying solely on individual foot timings, a fixed 0.3 s analysis window was defined around the foot of every cycle and used for correlation analysis, and the correlation coefficients from all 54 windows were averaged to reduce the influence of cycle-to-cycle variations.

The representative PTT from the whole recording was 0.055 s, corresponding to a PWV estimate of 8.7 m/s for the 0.48 m effective path length. The within-record bootstrap interval was 7.7–9.9 m/s, and the eight segment-wise estimates were 9.1 ± 1.8 m/s. These values are physiologically plausible for a young adult [30], but plausibility does not establish measurement accuracy. The 19.8% coefficient of variation of segment-wise PWV indicates that a 5 s segment was insufficient for a stable estimate with the present acquisition rate and handheld carotid unit; averaging over tens of seconds was required. Beyond this measurement variability, PWV in healthy young adults also varies over the course of a day [31], so an absolute value obtained in a single session should be interpreted with caution.

The temporal resolution of the recorded pulse-wave signals was limited by the mean 13.2 Hz acquisition rate, corresponding to a sampling interval of approximately 0.076 s. This interval was longer than the estimated PTT of 0.055 s, so no individual cycle could resolve the transit time directly; a sub-sample estimate was instead obtained from the peak location of a Gaussian function fitted to the correlation curve averaged over all cycles. The estimate also depends on the placement and length of the analysis window: windows extending beyond the carotid systolic peak include waveform regions whose morphology differs between the two sites and shorten the apparent transit time. This dependence is documented in Figure S1 of the Supplementary Materials; windows confined to the upstroke gave PWV values between approximately 7 and 12 m/s. A higher pulse-wave acquisition rate would provide finer temporal sampling and reduce reliance on interpolation for PTT estimation. Restricting the wavelength sweep to a smaller range would increase the peak time sensitivity in proportion to the reduction in sweep slope and could improve the equivalent strain resolution and the precision of peak localization. It would not, however, improve the native temporal sampling of the PTT measurement, for which a higher pulse-wave acquisition rate would be required separately. In addition, the effective path length was obtained by surface-distance subtraction, which approximates rather than measures the arterial path. A single FBG cannot separate strain from temperature. In the carotid unit, the measurement and reference gratings share the same elastomer body, so ambient and handle-borne thermal variation is expected to be largely common to the two channels and may therefore be partially suppressed by RFR together with the mechanical disturbance, although temperature was not measured independently in this study; because PTT is obtained from relative timing rather than from signal amplitude, slow baseline drift is expected to have less influence on PTT than on the signal baseline or amplitude, although this effect was not independently quantified. The wristband radial unit, however, has no reference grating and is therefore not thermally compensated, which would become significant for recordings substantially longer than the 41.8 s used here.

This preliminary experiment involved one participant and one recording; therefore, the findings cannot establish between-session repeatability, between-participant variability, or clinical accuracy. The reported segment analysis and bootstrap interval characterize only within-record variation. In addition, the carotid–radial path length was estimated by surface-distance subtraction, and the PTT and PWV estimates were not compared simultaneously with a reference device [3,5].

Future work should use higher-rate acquisition, repeated probe placement and measurements, standardized contact pressure and sensor positioning, a larger cohort, and simultaneous comparison with an established PWV instrument. Within the present limitations, the results demonstrate the technical feasibility of combining site-specific FBG sensing units, a mechanically decoupled carotid reference channel, and ensemble correlation analysis for simultaneous carotid–radial pulse-wave measurement and preliminary PWV estimation.

## Figures and Tables

**Table 1:** Comparison of RFR with baseline detrending and filtering applied to the same carotid measurement FBG signal. The signal quality index was calculated as in Equation (9) after the same mean subtraction and windowing; peak r, PTT, and PWV were obtained from the time shift analysis over all 54 cycles; segment CV is the coefficient of variation of the PTT estimates from eight non-overlapping 5 s segments.

Method	*R*_SQI_	Peak r	PTT (s)	PWV (m/s)	Segment CV (%)
None (raw signal)	0.17	0.848	0.048	9.94	46.7
Polynomial detrend	0.31	0.843	0.048	10.09	46.5
High-pass filter, 0.5 Hz	0.74	0.870	0.048	10.03	38.9
High-pass filter, 0.8 Hz	0.82	0.867	0.047	10.20	34.6
RFR (this work)	0.46	0.896	0.055	8.69	20.3

## Data Availability

The data supporting the findings of this study are available from the corresponding author upon reasonable request.

## Supplementary Materials

The following supporting information can be downloaded at https://www.mdpi.com/article/10.3390/s26216879/s1. Figure S1: Dependence of the estimated PTT and of the peak correlation coefficient on the offset and length of the analysis window.

## Abbreviations

The following abbreviations are used in this manuscript:

CV	Coefficient of variation
FBG	Fiber Bragg grating
FFT	Fast Fourier transform
MSA	Mean squared amplitude
PPG	Photoplethysmography
PTT	Pulse transit time
PWV	Pulse-wave velocity
RFR	Reference FBG regression
SD	Standard deviation
SQI	Signal quality index

## References

[1] Bramwell J.C., Hill A.V. (1922). The Velocity of Pulse Wave in Man. Proc. R. Soc. Lond. Ser. B Contain. Pap. Biol. Character.

[2] Laurent S., Cockcroft J., Van Bortel L., Boutouyrie P., Giannattasio C., Hayoz D., Pannier B., Vlachopoulos C., Wilkinson I., Struijker-Boudier H. (2006). Expert Consensus Document on Arterial Stiffness: Methodological Issues and Clinical Applications. Eur. Heart J..

[3] Townsend R.R., Wilkinson I.B., Schiffrin E.L., Avolio A.P., Chirinos J.A., Cockcroft J.R., Heffernan K.S., Lakatta E.G., McEniery C.M., Mitchell G.F. (2015). Recommendations for Improving and Standardizing Vascular Research on Arterial Stiffness: A Scientific Statement From the American Heart Association. Hypertension.

[4] Segers P., Rietzschel E.R., Chirinos J.A. (2020). How to Measure Arterial Stiffness in Humans. Arterioscler. Thromb. Vasc. Biol..

[5] Milan A., Zocaro G., Leone D., Tosello F., Buraioli I., Schiavone D., Veglio F. (2019). Current Assessment of Pulse Wave Velocity: Comprehensive Review of Validation Studies. J. Hypertens..

[6] Pereira T., Correia C., Cardoso J. (2015). Novel Methods for Pulse Wave Velocity Measurement. J. Med. Biol. Eng..

[7] Allen J. (2007). Photoplethysmography and Its Application in Clinical Physiological Measurement. Physiol. Meas..

[8] Benavides Cabrera L.S., Costa da Silva E., Costa Monteiro E. (2025). Highly Sensitive Pressure Transducer for Measuring Arterial Pulse Wave Velocity Based on Giant Magneto-Impedance Sensors. Sensors.

[9] Jannasz I., Sondej T., Targowski T., Mańczak M., Obiała K., Dobrowolski A.P., Olszewski R. (2023). Relationship between the Central and Regional Pulse Wave Velocity in the Assessment of Arterial Stiffness Depending on Gender in the Geriatric Population. Sensors.

[10] Diana G., Scardulla F., Puleo S., Pasta S., D’Acquisto L. (2025). Non-Invasive Estimation of Arterial Stiffness Using Photoplethysmography Sensors: An In Vitro Approach. Sensors.

[11] Lo Presti D., Massaroni C., Leitão C.S.J., Domingues M.D.F., Sypabekova M., Barrera D., Floris I., Massari L., Oddo C.M., Sales S. (2020). Fiber Bragg Gratings for Medical Applications and Future Challenges: A Review. IEEE Access.

[12] Hill K.O., Meltz G. (1997). Fiber Bragg Grating Technology Fundamentals and Overview. J. Light. Technol..

[13] Othonos A. (1997). Fiber Bragg Gratings. Rev. Sci. Instrum..

[14] Kersey A.D., Davis M.A., Patrick H.J., LeBlanc M., Koo K.P., Askins C.G., Putnam M.A., Friebele E.J. (1997). Fiber Grating Sensors. J. Light. Technol..

[15] Rao Y.-J. (1997). In-Fibre Bragg Grating Sensors. Meas. Sci. Technol..

[16] Lee B. (2003). Review of the Present Status of Optical Fiber Sensors. Opt. Fiber Technol..

[17] Canning J. (2008). Fibre Gratings and Devices for Sensors and Lasers. Laser Photonics Rev..

[18] Mishra V., Singh N., Tiwari U., Kapur P. (2011). Fiber Grating Sensors in Medicine: Current and Emerging Applications. Sens. Actuators A Phys..

[19] Roriz P., Frazão O., Lobo-Ribeiro A.B., Santos J.L., Simões J.A. (2013). Review of Fiber-Optic Pressure Sensors for Biomedical and Biomechanical Applications. J. Biomed. Opt..

[20] Mishra M., Sahu P.K. (2023). Fiber Bragg Gratings in Healthcare Applications: A Review. IETE Tech. Rev..

[21] Li Y., Wang B., Liu S., Gao M., Li Q., Chen C., Guo Q., Yu Y. (2024). Fiber Bragg Grating Pulse and Systolic Blood Pressure Measurement System Based on Mach–Zehnder Interferometer. Sensors.

[22] Wang Y.-J., Wang L. (2025). Blood Pressure and Heart Rate Measurements Using Fiber Bragg Grating Sensor with Optical Power Detection Scheme. Sensors.

[23] Umesh S., Goggi S.P., Ambastha S., Kalegowda A., Asokan S. (2015). Pulse Transit Time Differential Measurement by Fiber Bragg Grating Pulse Recorder. J. Biomed. Opt..

[24] Wang J., Wang Z., Zhang Z., Li P., Pan H., Ren Y., Hou T., Wang C., Kwong C.-F., Zhang B. (2024). Simultaneous Measurement of Local Pulse Wave Velocities in Radial Arteries Using a Soft Sensor Based on the Fiber Bragg Grating Technique. Micromachines.

[25] Widrow B., Glover J.R., McCool J.M., Kaunitz J., Williams C.S., Hearn R.H., Zeidler J.R., Dong E., Goodlin R.C. (1975). Adaptive Noise Cancelling: Principles and Applications. Proc. IEEE.

[26] Gagnon L., Cooper R.J., Yücel M.A., Perdue K.L., Greve D.N., Boas D.A. (2012). Short Separation Channel Location Impacts the Performance of Short Channel Regression in NIRS. NeuroImage.

[27] Yücel M.A., Selb J., Aasted C.M., Petkov M.P., Becerra L., Borsook D., Boas D.A. (2015). Short Separation Regression Improves Statistical Significance and Better Localizes the Hemodynamic Response Obtained by Near-Infrared Spectroscopy for Tasks with Differing Autonomic Responses. Neurophotonics.

[28] Elgendi M. (2016). Optimal Signal Quality Index for Photoplethysmogram Signals. Bioengineering.

[29] Heinzel G., Rüdiger A., Schilling R. (2002). Spectrum and Spectral Density Estimation by the Discrete Fourier Transform (DFT), Including a Comprehensive List of Window Functions and Some New Flat-Top Windows.

[30] Bia D., Zócalo Y. (2021). Physiological Age- and Sex-Related Profiles for Local (Aortic) and Regional (Carotid-Femoral, Carotid-Radial) Pulse Wave Velocity and Center-to-Periphery Stiffness Gradient, with and without Blood Pressure Adjustments: Reference Intervals and Agreement between Methods in Healthy Subjects (3–84 Years). J. Cardiovasc. Dev. Dis..

[31] Bodlaj G., Berg J., Biesenbach G. (2007). Diurnal Variation of Pulse Wave Velocity Assessed Non-Invasively by Applanation Tonometry in Young Healthy Men. Yonsei Med. J..

