# Supplementary material for: Reference-Assisted FBG Sensing for Simultaneous Carotid–Radial Pulse-Wave Measurement and PWV Estimation
Source: Sensors (Basel). 2026 Oct 30;26(21):6879. doi: 10.3390/s26216879 (PMC13766336; doi:10.3390/s26216879)
Supplement: Supplementary file 1 [file sensors-26-06879-s001.zip › sensors-4645502-supplementary.pdf]

Supplementary Materials

Reference-Assisted FBG Sensing for Simultaneous Carotid–Radial Pulse-Wave Measurement and PWV Estimation

Chang Hyun Park, Minju Jeong, Seongmun Jeong, Min Hur, Yoonji Lee, Hyung-Hoi Kim, Jeesu Kim, Chang-Seok Kim and Hwidon Lee

Manuscript ID: sensors-4645502

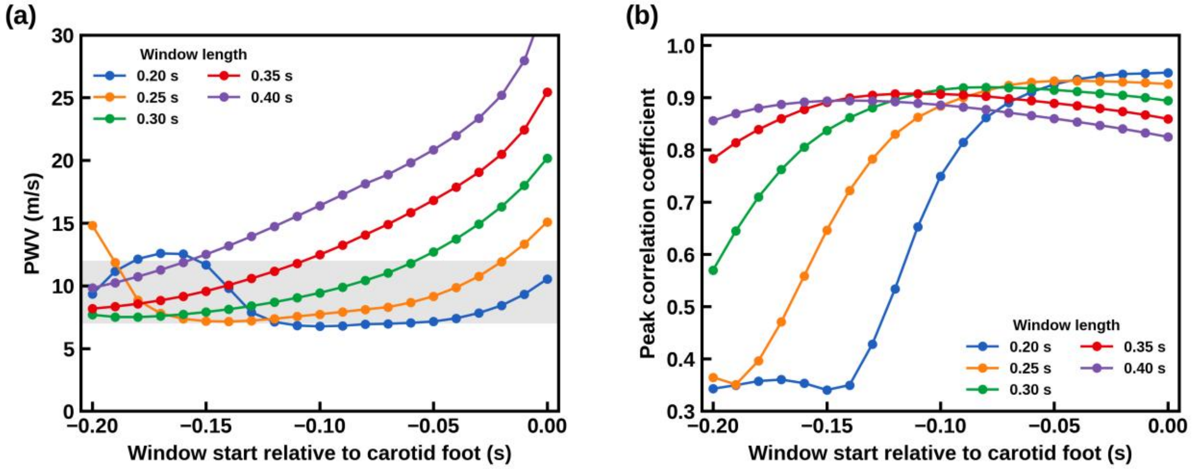

Figure S1. Sensitivity of the correlation-based transit-time estimate to the definition of the analysis window, evaluated over all 54 analysable cardiac cycles of the 41.8-s recording: (a) estimated pulse wave velocity and (b) peak correlation coefficient, as a function of the window start relative to the carotid pulse-wave foot, for window lengths of 0.20–0.40 s. The shaded band in (a) marks the 7–12 m/s range. Windows that close before the mean carotid systolic peak, which occurs 0.26 s after the foot, give estimates within this range, whereas windows extending beyond the systolic peak include waveform regions whose morphology differs between the carotid and radial sites and progressively shorten the apparent transit time. The window used in the main text starts 0.12 s before the foot and has a length of 0.30 s.

Note on the underlying analysis. For every combination of window offset and window length, the analysis described in Section 2.4.3 of the main text was repeated in full: a window of the specified offset and length was placed about the detected foot of each of the 54 cycles, 21 candidate time shifts from −0.10 to 0 s were applied to the radial signal in 0.005-s steps, the correlation coefficients were averaged over all cycles, and a Gaussian function was fitted to the resulting curve. The analysis uses only the peak-time shift $\Delta\tau p$ and is therefore independent of the strain calibration.
